# 4D flow cardiovascular magnetic resonance consensus statement

**DOI:** 10.1186/s12968-015-0174-5

**Published:** 2015-08-10

**Authors:** Petter Dyverfeldt, Malenka Bissell, Alex J. Barker, Ann F. Bolger, Carl-Johan Carlhäll, Tino Ebbers, Christopher J. Francios, Alex Frydrychowicz, Julia Geiger, Daniel Giese, Michael D. Hope, Philip J. Kilner, Sebastian Kozerke, Saul Myerson, Stefan Neubauer, Oliver Wieben, Michael Markl

**Affiliations:** Division of Cardiovascular Medicine, Department of Medical and Health Sciences, Linköping University, Linköping, Sweden; Center for Medical Image Science and Visualization, Linköping University, Linköping, Sweden; Division of Cardiovascular Medicine, Radcliffe Department of Medicine, University of Oxford Centre for Clinical Magnetic Resonance Research, Oxford, UK; Department of Radiology, Northwestern University, Chicago, USA; Department of Medicine, University of California San Francisco, San Francisco, CA United States; Department of Clinical Physiology, Department of Medical and Health Sciences, Linköping University, Linköping, Sweden; Department of Radiology, University of Wisconsin, Madison, Wisconsin USA; Klinik für Radiologie und Nuklearmedizin, University Hospital Schleswig-Holstein, Campus Lübeck, Lübeck, Germany; Department of Radiology, University Children’s Hospital Zurich, Zurich, Switzerland; Department of Radiology, University Hospital of Cologne, Cologne, Germany; Department of Radiology, University of California San Francisco, San Francisco, CA United States; NIHR Cardiovascular Biomedical Research Unit, Royal Brompton and Harefield NHS Foundation Trust, National Heart and Lung Institute, Imperial College, London, UK; Institute for Biomedical Engineering, University and ETH Zurich, Zurich, Switzerland; Department of Medical Physics, University of Wisconsin, Madison, Wisconsin USA; Department of Biomedical Engineering, Northwestern University, Chicago, IL USA

**Keywords:** 4D Flow CMR, 4D Flow MRI, Phase-contrast magnetic resonance imaging, MR flow imaging, Hemodynamics, Flow visualization, Flow quantification, Recommendations, Clinical, Cardiovascular

## Abstract

Pulsatile blood flow through the cavities of the heart and great vessels is time-varying and multidirectional. Access to all regions, phases and directions of cardiovascular flows has formerly been limited. Four-dimensional (4D) flow cardiovascular magnetic resonance (CMR) has enabled more comprehensive access to such flows, with typical spatial resolution of 1.5×1.5×1.5 – 3×3×3 mm^3^, typical temporal resolution of 30–40 ms, and acquisition times in the order of 5 to 25 min. This consensus paper is the work of physicists, physicians and biomedical engineers, active in the development and implementation of 4D Flow CMR, who have repeatedly met to share experience and ideas. The paper aims to assist understanding of acquisition and analysis methods, and their potential clinical applications with a focus on the heart and greater vessels. We describe that 4D Flow CMR can be clinically advantageous because placement of a single acquisition volume is straightforward and enables flow through any plane across it to be calculated retrospectively and with good accuracy. We also specify research and development goals that have yet to be satisfactorily achieved. Derived flow parameters, generally needing further development or validation for clinical use, include measurements of wall shear stress, pressure difference, turbulent kinetic energy, and intracardiac flow components. The dependence of measurement accuracy on acquisition parameters is considered, as are the uses of different visualization strategies for appropriate representation of time-varying multidirectional flow fields. Finally, we offer suggestions for more consistent, user-friendly implementation of 4D Flow CMR acquisition and data handling with a view to multicenter studies and more widespread adoption of the approach in routine clinical investigations.

## Introduction

Pulsatile blood flow through the cavities of the heart and great vessels is multidirectional and multidimensional. However, access to all the directions, regions and phases of such flows has been limited with cardiovascular magnetic resonance (CMR) as well as other imaging modalities. Four-dimensional (4D) flow CMR has been developed to attain more comprehensive access to blood flow through the heart and large vessels [1–4]. This unique technique enables a wide variety of options for visualization and quantification of flow, ranging from basic aspects such as flow volume and peak velocity to more advanced features such as the estimation of hemodynamic effects at the vessel wall and myocardium, as well as visualization of flow pathways in the heart and great vessels.

“4D Flow CMR” refers to phase-contrast CMR with flow-encoding in all three spatial directions that is resolved relative to all three dimensions of space and to the dimension of time along the cardiac cycle (3D + time = 4D). For concise description and unification of nomenclature we recommend the use of the term “4D Flow CMR” or “4D Flow MRI” as will be used throughout the document. For methodological clarification, we recommend that a full-length description such as “three-dimensional (3D) cine (time-resolved) phase-contrast CMR with three-directional velocity-encoding” is included in the methods section of reports that employ this technique.

In 2012, leading teams in 4D Flow CMR started meeting on a regular basis to discuss the state of the technique and directions for future work. This group consists of medical physicists, physicians, and biomedical engineers who have been active in cardiovascular 4D Flow CMR research. As the field has grown, the list of invitees was extended by personal acquaintance and availability to join regular meetings. Soon, the need to find a uniform basis for terminology, potential clinical applications, and various technical aspects regarding data acquisition, data processing, visualization, and quantification became apparent. Therefore, we started a consensus initiative during the 2nd biannual 4D Flow Workshop held in Oxford, UK, in September 2013 to summarize our discussions.

This consensus statement aims to assist the understanding of the acquisition, analysis, and possible clinical applications of 4D Flow CMR in the heart and great vessels (aorta, pulmonary arteries), including all the steps involved in a 4D Flow CMR study. We will also discuss research and development goals that have yet to be achieved, in order to address current limitations and ensure data reliability and validity.

The consensus statement is based on published data and the shared experience of the 4D Flow CMR consensus group. The authors understand the manuscript as a state-of-the-art summary on the acquisition or analysis of 4D Flow CMR that creates a basis for future multivendor and multicenter research, can serve as a reference to established research, and provide guidance to researchers and clinicians new to the field.

### Clinical and scientific significance

Flow assessment has long been used in the evaluation of cardiovascular disease. In recent decades, largely due to the advent of multidimensional flow imaging and computational fluid dynamics (CFD), the importance of improving our understanding of physiological and pathophysiological blood flow conditions is increasingly acknowledged [5, 6].

The most common clinical tool for cardiovascular flow assessment is Doppler echocardiography, which can measure the blood flow velocity component in the direction of the ultrasound beam or provide a 2D visualization of one-directional blood flow velocities using the color Doppler mode. Doppler echocardiography is often used to assess peak and mean velocities in the aorta and pulmonary artery for calculation of peak and mean pressure drops, known as gradients, via the simplified Bernoulli equation [7, 8]. This approach, however, is limited by the constraints associated with echo-Doppler imaging, which include variable velocity assessment (due to beam alignment), limited acoustic window, and operator expertise [9–11]. Further, the calculation of mean velocities and net flow is often based on assumptions regarding the underlying flow profile and vessel cross-sectional area which may results in inaccurate flow quantification in the presence of complex flow and/or vessel geometry.

The most common CMR flow imaging technique is 2D cine phase contrast (PC) CMR with velocity-encoding in a single direction (2D cine PC-CMR) [12–19]. The single velocity-encoding direction is typically selected perpendicular to the 2D plane, which enables calculation of the volume of flow passing through the plane. 2D cine PC-CMR is arguably the gold-standard for flow volume quantification. The formerly widely used, but invasive thermodilution technique for flow quantification, is subject to inaccuracies due to underlying assumptions. Unlike Doppler echocardiography or 2D cine PC-CMR, 4D Flow CMR acquisition includes measurements representing all directions and spatial regions of flow within the boundaries of the volume imaged. Although methodologically different, computational fluid dynamics (CFD) is comparable in terms of multidirectional, volumetric representation [20–25]. Flow fields can potentially be calculated by CFD to high spatial and temporal resolution [26–28]. However, CFD requires accurate definition of geometrical and physiological boundary conditions and the ability of the computed flow fields to represent reality depends on the precision of the boundary conditions and the validity of underlying assumptions. For these reasons, CFD is currently not used in clinical decision-making. The relatively direct, voxel by voxel measurements of velocity provided by 4D Flow CMR can be complementary to the higher resolution velocity fields computed by CFD.

### Clinical utility

CMR-based flow volume quantification is routinely used at many institutions to estimate shunt flows, regurgitant flows, collateral flows, etc. [29, 30]. These diagnostic tests are primarily based on 2D cine PC-CMR. A large number of studies across different institutions and MR-systems have demonstrated that 4D Flow CMR permits flow volume quantification that is comparable to 2D cine PC-CMR [31–39] and has good scan-rescan repeatability [32, 40, 41]. A recent study that assessed Qp/Qs ratios in intracardiac shunts reported that flow volumes are underestimated compared to 2D cine PC-CMR but that the Qp/Qs ratios were not different [42]. 2D cine PC-CMR has been hampered by artifacts such as background phase offsets which can lead to errors inflow volume measurements. These issues are shared by 4D Flow CMR and proper measures to compensate for them are necessary. However, flow volume quantification with 4D Flow CMR has several advantages when compared to 2D cine PC-CMR. 4D Flow CMR permits investigation of the internal consistency of the data by employing the ‘conservation of mass’ principle (e.g. Qp/Qs within the same dataset). This important feature lends itself well to standardization of data quality assurance and will be discussed later. Investigators have used this feature and demonstrated that flow volume measurements with 4D Flow CMR have good internal consistency [32, 38, 41–49].

Another advantage of 4D Flow CMR is the retrospective placement of analysis planes at any location within the acquisition volume. While standard 2D cine PC techniques can easily be applied during a single breath hold, 4D Flow CMR on the other hand, offers the ability to retrospectively calculate blood flow through any planes of interest across the 3D volume. Despite longer scan times, 4D Flow CMR allows easy scan prescription (positioning of a single 3D volume) compared to the need to predetermine and accurately locate all relevant planes of 2D acquisitions. This may be especially advantageous in cases where multiple 2D cine PC-CMR scans would be needed [36]. In these situations, 4D Flow CMR may even be faster than prescribing and scanning a series of 2D cine breath-held PC CMR acquisitions, enabling a reduced period of anesthesia for younger children or of scan time in decompensated patients. Further, the option of valve tracking may improve assessment of flow through heart valves [43]. Compared to 2D cine PC-CMR, 4D Flow CMR measures velocity in all spatial directions and has superior spatial coverage and may therefore also be better at capturing the peak velocity of a stenotic jet [37]. However, one recent study suggested that the peak flow rate was lower for 4D compared to 2D flow [35]. These findings may in part be explained by relatively low temporal resolution (50–55 ms). Similarly, another recent study with 46 ms temporal resolution obtained smaller net flow volumes with 4D compared to 2D flow CMR [42]. Larger, preferably multicenter, studies with optimized protocols would be helpful to establish the comparability between 2D and 4D Flow CMR, as well as the spatial and temporal resolution needed for different applications.

In addition to the flexible retrospective quantification of conventional flow parameters, 4D Flow CMR allows for the visualization of multidirectional flow features and alterations of these associated with cardiovascular disease [50–53]. Previously reported results include the application of 4D Flow CMR for the analysis of blood flow in the ventricles [54–63] and atria [64–67] of the heart, heart valves [3, 43, 68, 69], aorta [41, 69–82], main pulmonary vessels [83–86], carotid arteries [87–90], large intracranial arteries and veins [91–98], arterial and portal venous systems of the liver [46, 85, 99–101], peripheral arteries [102] and renal arteries [103, 104]. The intuitive flow visualizations that 4D Flow CMR offers have already found utility in several clinical studies. For example, time-resolved visualizations of blood flow have been used clinically to identify flow directionality and areas of flow acceleration in visceral abdominal blood flow [105–107]. In addition, a number of studies have shown that visualization of aortic blood flow can be helpful to quickly identify regions with high velocity flow close to the vessel wall that may indicate altered fluid mechanical effects on the vessel wall [69, 74, 108–110]. Finally, there are promising applications in complex congenital heart disease [39, 85, 111, 112]. While these examples are promising and illustrate the potential of 4D Flow analysis to better understand complex hemodynamic patterns, the clinical utility needs further evaluation in larger prospective and multi-center trials. For a more detailed overview of recent 4D Flow CMR developments and its use for 3D flow visualization and quantification throughout the human circulatory systems the reader is referred to a number of recently published review articles [113–118].

### Research utility

4D Flow CMR has made it possible to investigate in-vivo cardiovascular flow fields more comprehensively than was previously possible. Multidisciplinary research teams are using the technique to 1) address gaps in the understanding of cardiovascular physiology and pathophysiology, 2) better understand the impact of hemodynamics on the heart and vasculature, 3) delineate further to what degree alterations of flow predispose to or result from cardiovascular disease processes such as remodeling, and 4) assess the degree to which physiological flow and pressure profiles have been restored following interventional or surgical procedures. Thus, by affording visualization and quantification of flow parameters ranging from conventional parameters such as flow volume and regurgitant fraction to more advanced parameters such as flow energetics and shear stress, there are several applications where 4D Flow CMR has significant potential for advancing our knowledge and assessment of the cardiovascular system. For example, 4D Flow CMR has been used to demonstrate separation of blood that transits heart chambers according to compartmental origin and fate, retrograde flow embolization pathways from the descending aorta to the brain, and associations of valve outflow jet patterns with aortopathy [56, 74, 82, 109, 119–124]. In addition, the technique can be used to derive new physiologic and pathophysiologic hemodynamic parameters such as such as wall shear stress [125–127], pressure difference [103, 128–131], pulse wave velocity [132, 133], turbulent kinetic energy [134–137], and others [57, 58, 122, 138–141] for more differentiated characterization of cardiovascular pathophysiology beyond simple measures of flow. The majority of these in-vivo hemodynamic measures cannot be assessed non-invasively with any other imaging technique. In all areas, further studies are required to assess the clinical impact of these measurements.

### Consensus recommendations

This section provides recommendations for the use of 4D Flow CMR for 3D flow visualization and quantification of flow volume, retrograde flow and peak velocity in the heart and large vessels (aorta, pulmonary arteries). We focus on basic flow visualization and standard CMR parameters which can most easily been incorporated into routine clinical application. More advanced parameters such as pressure difference mapping, wall shear stress and turbulent kinetic energy are not addressed in this section but will be discussed in the future work section below. Table 1 lists analysis parameters and visualizations that are recommended for different clinical indications. The recommendations for quantification are based on the literature described in the “Clinical utility” section above. We emphasize that the amount of supporting literature is smaller for flow visualization compared to quantification and thus the recommendations are primarily based on consensus discussions.

**Table 1:** Recommended 4D Flow CMR analysis for different clinical indications - all aspects below can be derived from a single acquisition. For a comprehensive overview of 4D Flow CMR quantification and visualization methodology including additional references please see recently published review articles [113–118]

Clinical indication	Quantification	Visualization^a^
Heart valve disease (stenosis, regurgitation)	Flow volume	• Identification of regurgitant and stenotic jets using streamlines and pathlines
	• Regurgitant flow volumes & fraction	• Peak velocity location by systolic streamlines or maximum intensity projections of speed images
	Peak velocity	• Outflow patterns using streamlines
	• Estimated pressure gradients with modified Bernoulli equation	• Time course of flow curve
Shunts and collateral vessels (Ventricular-septal defect, atrial-septal defect, fistulae)	Flow volume	• Identification of shunt flow and flow directionality using pathlines
	• Shunt flow volume	
	• Qp/Qs	
Complex congenital heart disease (e.g. single ventricle physiology, Fontan circulation, Fallot’s tetralogy),	Flow volume	• Flow directionality using pathlines
	• Regurgitant flow volumes & fraction	• Shunt flow using pathlines
	• Flow distribution (e.g. left vs right pulmonary artery, relative SVC/IVC flow)	• Flow connectivity and distribution using pathlines
	• Collateral flow volume	
	Peak velocity	
Aortic disease (aneurysm, coarctation, dissection)	Flow volume	• Peak velocity location by systolic streamlines or maximum intensity projections of speed images
	• Regurgitant flow volumes & fraction	• Identification of flow in false lumen and potential entry/exit sites
	• Relative flows in true & false lumen	• Identification of highly disrupted flow patterns (likely to reduce forward flow) in tortuous aortic conditions
	Peak velocity	

^a^The amount of supporting literature is smaller for visualization compared to quantification

**Fig. 1:**
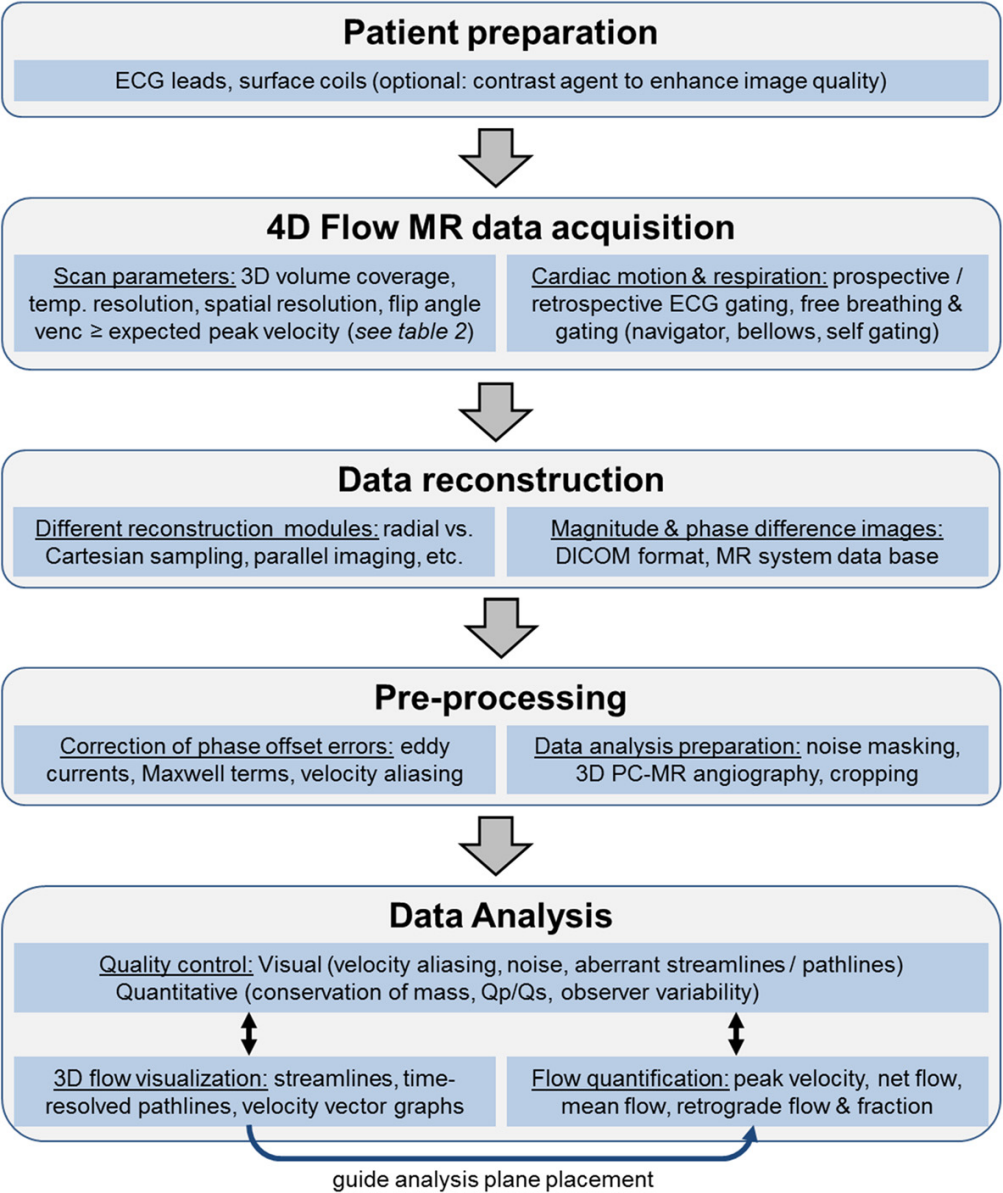
Recommended workflow for clinical application of 4D Flow CMR with the main components of 1) patient preparation, 2) data acquisition in the magnet, 3) data reconstruction, 4) pre-processing of the reconstructed data, and 5) data analysis

Several steps are required to assess blood flow with 4D Flow CMR including proper patient preparation, choice of acquisition parameters, and data conditioning through pre-processing, and data analysis. We suggest a structured workflow for data acquisition and processing as shown in Fig. 1.

### Patient preparation

4D Flow CMR requires a reliable ECG trace with detectable R-wave to ensure consistency between RR-intervals. Standard ECG positioning applies. For aortic flow assessment it is important that the surface coils are positioned high enough to also fully encompass the aortic arch, which can be quite high in some aortic pathologies. 4D Flow CMR scans can be relatively long and it is useful to inform the patient about this prior to starting the scan in order to minimize discomfort.

### 4D flow CMR data acquisition

#### PC-MR signal and use of contrast agents

4D Flow CMR employs spoiled gradient echo sequences with short TR for rapid imaging. As such, the signal magnitude from the blood is weighted inversely with the T1 relaxation time. This allows for the generation of PC angiograms without the need for an external contrast agent [107, 142]. Although 4D Flow CMR does not require any contrast agents, it is often used as part of a comprehensive CMR study that does requires the use of T_1_ shortening gadolinium-based contrast agents, for example for perfusion MRI or late gadolinium enhancement imaging. In such cases, acquiring the 4D Flow CMR data after the study that requires contrast administration takes advantage of the enhanced signal-to-noise ratio (SNR) and thus velocity to noise ratio (VNR) as well as contrast between blood and surrounding tissue [107, 143, 144]. However, contrast agents that wash out during the 4D Flow scan can result in time-varying blood T1 times and the effects of this variability on PC-CMR velocity data is not fully known. As a result, the effect of contrast agents depend on the type (extravascular vs. intravascular) and SNR, VNR can vary depending on the timing of the contrast agent administration.

#### Scan parameters

Table 2 provides an overview of the most important scan and reconstruction parameters and lists recommended values for each of them. The recommendations are primarily based on the experience of the authors of this consensus document and the literature that has investigated 4D Flow-based flow volume quantification [31–38, 40–48]. We propose this list of parameter settings as a baseline 4D Flow CMR protocol against which alterative protocols can be compared. Specialized applications such as measurements in a paediatric population or analysis of advanced flow parameters may require optimized parameter choices. Table 2 also indicates the potential advantages of ‘ideal’ parameters, the factors that may prevent their achievement and a value of the parameter that has been shown to be practicable and sufficient for most flow quantification in 4D Flow CMR covering the heart and/or large vessels (aorta, pulmonary artery) in healthy adults. The main technical goal of a 4D Flow CMR acquisition is accuracy. This is largely determined by sufficient spatial and temporal resolution, and adequate SNR (hence VNR). The accuracy is also affected by artefacts. The practical achievability of the optimum parameter values is mostly hampered by total scan time, the methods availability and validity as well as system imperfections.

**Table 2:** 4D Flow CMR scan parameters

	Ideally	Reason	Limiting factor	Consensus value
Acquisition Parameters				
Field of view	Max	SNR, coverage	Scan time, system imperfections	Cover region of interest
Spatial resolution^a^	Maximum, at least 5–6 voxels across vessel diameter of interest^b^, isotropic resolution.	Accuracy	Scan time, SNR	<2.5×2.5×2.5 mm^3^ for aorta or pulmonary artery
				<3.0×3.0×3.0 mm^3^ for whole heart and greater vessels
Velocity encoding timing (beat- vs. TR-interleaved)	TR-interleaved	Avoid inter-cycle variability	Temporal resolution	TR-interleaved
k-space segmentation factor	1	Accuracy (temporal resolution)	Scan time	2
Temporal resolution^c^	Max	Accuracy	Scan time	<40 ms
ECG synchronization^d^	Retrospective	Cover entire ECG cycle, avoid sequence interruption	Reconstruction complexity	If available: retrospective
				Else: Prospective^e^
Respiratory motion compensation^f^	100 % acceptance, motion correction	Scan time, reduction of breathing artifacts	Reconstruction complexity, robustness, breathing artefacts (ghosting and blurring)	If available: Leading or trailing MR navigator on liver/diaphragm interface, 6 mm window size, typically resulting in 50 % acceptance rate.
				Otherwise: Bellows with 50 % acceptance rate.
Partial k-space coverage in phase- and slice-encoding directions	Full k-space coverage	SNR, resolution	Scan time	If available: Elliptical k-space
				Otherwise: Half scan 75 % × 75 % (y × z)
Flip Angle^g^	Ernst angle: α = acos(e^-TR/T1^)	SNR	Contrast vs. SNR	Ernst angle
Parallel Imaging	No parallel imaging	SNR	Scan time	R = 2-3 (depends on #channels in coil array)
*k-t* undersampling^h^	No *k-t* under sampling	SNR	Scan time	If available: R = 4-5
Venc	Maximum expected velocity, multiple vencs	VNR, avoid aliasing	Scan time	Single venc, 10 % higher than maximum expected velocity
Postprocessing Parameters				
Maxwell correction	Yes	Accuracy		Yes
Eddy current correction	Yes	Accuracy	Different methods and their validity and robustness	Yes
Phase unwrapping	Yes	Accuracy	Different methods and their validity and robustness	Yes
Gradient non-linearity correction	Yes	Accuracy	Availability	If available

^a^Always indicate the effectively acquired resolution in combination with the interpolated resolution

^b^Studies have demonstrated that 5–6 voxels across the vessel diameter is sufficient for flow volume quantification [165]

^c^Always indicate the effectively acquired resolution. If a temporal interpolation is performed, also indicate the interpolated temporal resolution along with the interpolation method used

^d^So called self-gating techniques have been evaluated and may become an alternative to the ECG [32]

^e^For prospective gating, analyses that involve integration over the whole cardiac cycle needs to be accompanied with a description of how the incomplete temporal coverage was handled

^f^Different types of respiratory navigators exist; variants include approaches that allow less motion in the central parts of k-space. Always describe the method that has been used and indicate the mean navigator efficiency in percent as well as the navigator acceptance window in mm. For fix window sizes and no k-space reordering, 6 mm navigator window is recommended, and this typically results in 50 % navigator efficiency

^g^The SNR is strongly dependent on the in-flow effect, therefore the flip angle can be and is often chosen higher than the Ernst angle. When using contrast agents, the Ernst angle further increases (due to lower T_1_)

^h^ *k-t* undersampling factor 4–5 in combination with conventional parallel imaging factor 2–3 is not recommended

4D Flow CMR requires the user to define an upper velocity limit, termed the velocity encoding range (venc), similar to 2D cine PC-CMR techniques. Venc is defined as the (positive or negative) velocity that gives a phase shift of π radians. Since phase is a cyclic entity, phase shifts greater than π radians result in velocity aliasing, which are visible as phase wraps in flow images. Higher venc results in lower VNR. We recommend choosing a venc slightly greater than the maximum velocity expected in the territory of interest. In stenotic and regurgitant flows, a multi-venc approach can be useful.

The total scan time available for adding 4D Flow CMR to a routine clinical CMR exam is often the most important limiting factor. If the total 4D Flow scan time is limited, e.g. not more than 5–8 min, the following trade-offs may be useful:

1. Acquire free-breathing 4D Flow CMR without respiratory gating to increase scan efficiency (studies have demonstrated reasonably accurate flow volume quantification without compensation for respiratory motion) [32, 34].
2. Reduce temporal resolution by increasing the k-space segmentation factor to 3. This decreases the temporal resolution from approximately 40 ms to 60 ms and may result in reduced accuracy of peak velocity and flow volume quantification.
3. Reduce spatial resolution and SNR by acquiring 65 % × 65 % of k_y_ and k_z_ phase encoding lines

Employing these parameter adjustments can result in a substantial reduction of scan time. However, these changes will result in decreased spatiotemporal resolution and SNR and increased artifacts, which negatively impact flow quantification and visualization accuracy. Deviations from a validated standard protocol should be followed up by additional quality control.

In order to achieve comparability between different studies and to facilitate reproducibility of previously published work, a crucial requirement is the inclusion of all major scan and post-processing parameters in published reports. We recommend listing all scan parameters included in Table 2, and we encourage authors to specify the employed flow-encoding scheme, such as symmetric, asymmetric, or Hadamard 4-point encoding, 5-point-encoding, multipoint encoding etc. [137, 145–147]. The total scan time should be listed as the total scan time including respiratory gating efficiency or as the total scan time excluding navigator efficiency in combination with the respiratory gating efficiency.

#### Data pre-processing

4D Flow CMR data processing usually involves the use of automated or semi-automated corrections of known artefacts and often requires calculation of a geometric representation of the underlying 3D cardiac or vascular geometry through segmentation. Several sources of error can compromise 4D Flow CMR analysis and need to be addressed prior to flow quantification and visualization. Similar to 2D cine PC-CMR, the major sources of errors include eddy current effects [148], concomitant gradient field effects (Maxell terms) [149], gradient field non-linearity [150, 151], and phase wraps resulting in velocity aliasing [152, 153]. Correction strategies have been presented in the literature and should be applied and evaluated to ensure accurate flow quantification and visualization [148–151, 154]. Investigators have also explored various types of image enhancement methods (noise filtering, divergence free corrections, etc.) to improve data quality. The use of such methods should be clearly reported in manuscripts, as they can also affect data quality negatively. Details and recommendations for the most common types of data processing are provided below. We emphasize that optimal approaches for data processing, especially corrections for background phase offsets, may vary between MR systems, sequences, protocols and applications.

#### Background phase offsets, concomitant gradient fields

Concomitant gradient fields, also referred to as Maxwell fields, lead to spatially varying background phase offsets in any type of PC-CMR acquisition. Correction factors for the concomitant gradient field correction can be directly derived from the gradient waveforms used for the data acquisition [149]. This correction scheme is implemented on MR systems as part of the standard PC-CMR image reconstruction engine.

#### Background phase offsets, Eddy currents

The switching of time-varying magnetic field gradients result in changes in magnetic flux which in turn induce eddy currents in the conducting parts of the scanner system. These eddy currents alter the strengths and durations of the desired gradients and thus result in spatially and temporally varying phase offsets in any type of PC-CMR [155, 156]. Modern MR scanners have pre-emphasis systems that adjust the gradient waveforms by incorporating predictions of eddy currents effects. However, not all eddy current effects can be compensated for and there currently is no definite solution to remove all eddy current induced background phase offsets. We recommend the approach of fitting polynomials through the phase of tissue known to be static [148]. It should be noted that the order of the polynomial and the approach to detect static tissue may be vendor, sequence, and application specific. Assessment of heart-phase dependent differences is recommended.

#### Phase wraps, velocity aliasing

Blood flow velocities that exceed the velocity sensitivity (venc) value result in velocity aliasing, or phase wraps. We recommend that the venc is set higher than the maximum expected velocity. However, such a venc setting can cause insufficient VNR in interesting flow regions with low velocity. Also, it is not always possible to predict the maximum velocity. We therefore recommend the use of a phase-unwrapping algorithm. The phase-unwrapping algorithm should be robust and not risk introducing additional errors. Identification of abrupt phase shifts in the temporal domain is a commonly used approach [153]. It should be noted that the visual perception and optimal phase-unwrapping strategies are different for different flow-encoding schemes.

#### Phase-Contrast Magnetic Resonance Angiography (PC-MRA)

4D Flow CMR data can be used to derive time-averaged 3D phase-contrast MR angiography (PC-MRA) based on the combination of velocity and magnitude data [142, 143, 157, 158]. The 3D PC-MRA can be used to guide anatomic orientation for flow visualization and regional flow quantification.

### Data analysis

#### Flow visualization

We generally recommend users of 4D Flow CMR to engage in visualizations and learn to interact with the data. Multiple options for the visualization of volumetric, time-resolved velocity vector fields on a 2D screen exist and none is entirely representative of the rich underlying data. It is a matter of choosing the proper visualization approach or combination of approaches that best address a particular question.

**Fig. 2:**
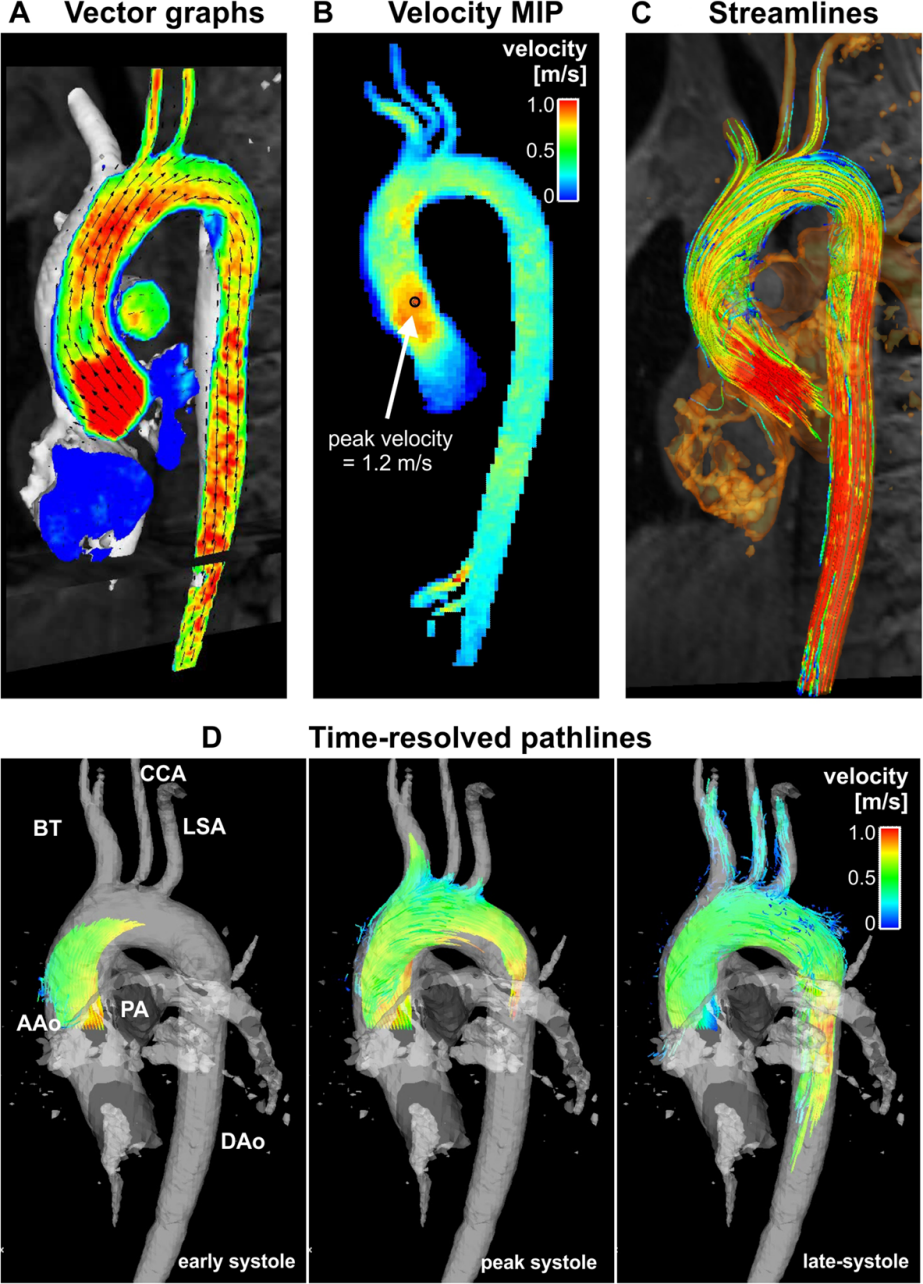
Examples of 4D Flow CMR visualization techniques. All examples are based on data acquired in the aorta of a healthy volunteer. In these examples, flow visualization is overlaid onto a segmentation of the aorta. **a** An oblique slice that transects the aorta has been color-coded by flow speed and combined with a graph of velocity vectors which here displays the speed and direction of blood velocity in black arrows at a coarser grid than the acquired voxels. This type of visualization provides a quick overview of velocity fields. **b** A maximum intensity projection (MIP) image of flow speed permits identification of areas of elevated velocity and the point of peak velocity while displaying the peak velocities of the whole volume projected onto this single slice image. **c** Streamlines are instantaneously tangent to the velocity vector field and are useful to visualize 3D velocity fields at discrete time points. Here, the peak systolic velocity field is shown. **d** Pathlines are the trajectories that massless fluid particles would follow through the dynamic velocity field. Pathlines are suitable for studies of the path of pulsatile blood flow over time. This example shows pathlines emitted from a plane in the ascending aorta at the onset of systole and traced to early systole (left), peak systole (middle) and late systole (right). All figures have been color-coded based on flow speed using the same color-window settings according to the scale shown in (**b**) and (**d**). In **a**, **c** and **d**, the visualizations have been combined with a PC-MRA isosurface which has been derived from the 4D Flow CMR data

**Fig. 3:**
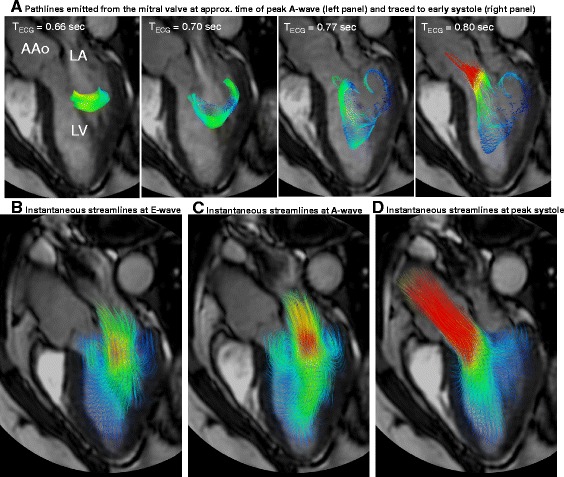
Examples of 4D Flow CMR visualization techniques, demonstrated on intracardiac flow data acquired in a healthy volunteer. In these examples, flow visualization is overlaid onto a 2D bSSFP acquisition in a three-chamber view. **a** Pathlines are the trajectories that massless fluid particles would follow through the dynamic velocity field and are suitable for studies of the path of pulsatile blood flow over time. Here, the transit of blood through the left ventricle (LV) is shown by pathlines emitted from the mitral valve at the time point of peak A-wave and traced to the time point of early systole systole. The timing of the ECG (T_ECG_) is included for reference. **b-d** Streamlines are instantaneously tangent to the velocity vector field and are useful to visualize 3D velocity fields at discrete time points. Here, streamlines generated in a long-axis plane show parts of the intracardiac velocity field at the time points of **b** peak early filling (E-wave), **c** peak late filling (A-wave), and **d** peak systole

Visualization techniques commonly used with 4D Flow CMR include vector maps, streamlines and pathlines as well as maximum intensity projections, isosurfaces and volume renderings (see Figs. 2 and 3) [50, 51, 54, 159]. The choice of one technique over another, and the choice of a color map, depends on the application in question, the display medium and the time available for processing, among other factors. Visualization of 4D Flow data can be time-consuming and often benefits from informed user interaction. Interactive user-guided visualizations are valuable for generation of flow-based hypotheses. Efforts toward more standardized automated flow visualization approaches could be helpful in certain applications and would minimize operator-dependent variation, although users should understand the principles, strengths and limitations of different techniques. Further descriptions of the various approaches and their applications can be found in recent review articles [113–118, 159, 160].

Relatively thorough interrogation of a flow field can be achieved by interactively browsing through the slices of a 4D dataset using vector map displays and/or color coding according to speed (magnitude of the velocity vector) (Fig. 2a) or by creating a maximum ‘intensity’ projection (MIP) for speed (Fig. 2b). Instantaneous streamlines can be used to represent the directions of flow throughout a cavity or vessel at a given cardiac phase. Comparable instantaneous visualizations are achieved by 3D velocity vector maps, which display the magnitude and direction of blood velocity of each voxel. Instantaneous streamlines are traces through a 3D velocity field, parallel to the velocity vectors at all spatial points along their length. They represent a specific temporal phase (see Fig. 2c and Fig. 3c-e). An instantaneous streamline does not, in a pulsatile flow field, correspond with the path traveled by any given blood cell [161]. In contrast, pathlines (Fig. 2d and Fig. 3a-b), follow the paths of virtual massless particles. Streamlines and pathlines of a pulsatile flow field differ from each other, and each should be interpreted accordingly. To explore the path of blood through space and time, pathlines are likely to be more telling, whereas instantaneous streamlines would be more suitable for depictions of instantaneous flow features. Inclusion of the adjective “instantaneous” (instantaneous streamlines) helps to avoid confusion.

Many analysis parameters, such as flow speed, vorticity and turbulent kinetic energy, are scalar fields that can be visualized using MIP images or isosurface and volume rendering techniques. Isosurfaces and volume renders can be combined with vector graphs, streamlines or pathlines to create visualizations of multiple parameters. 4D Flow CMR visualizations may also be fused with other types of MR images, such as contrast-enhanced MR angiography and balanced steady-state free-precession (bSSFP) cine images to display anatomy. Such combinations can provide additional integration of cardiovascular morphology and function (see example in Fig. 3).

#### Flow quantification

**Fig. 4:**
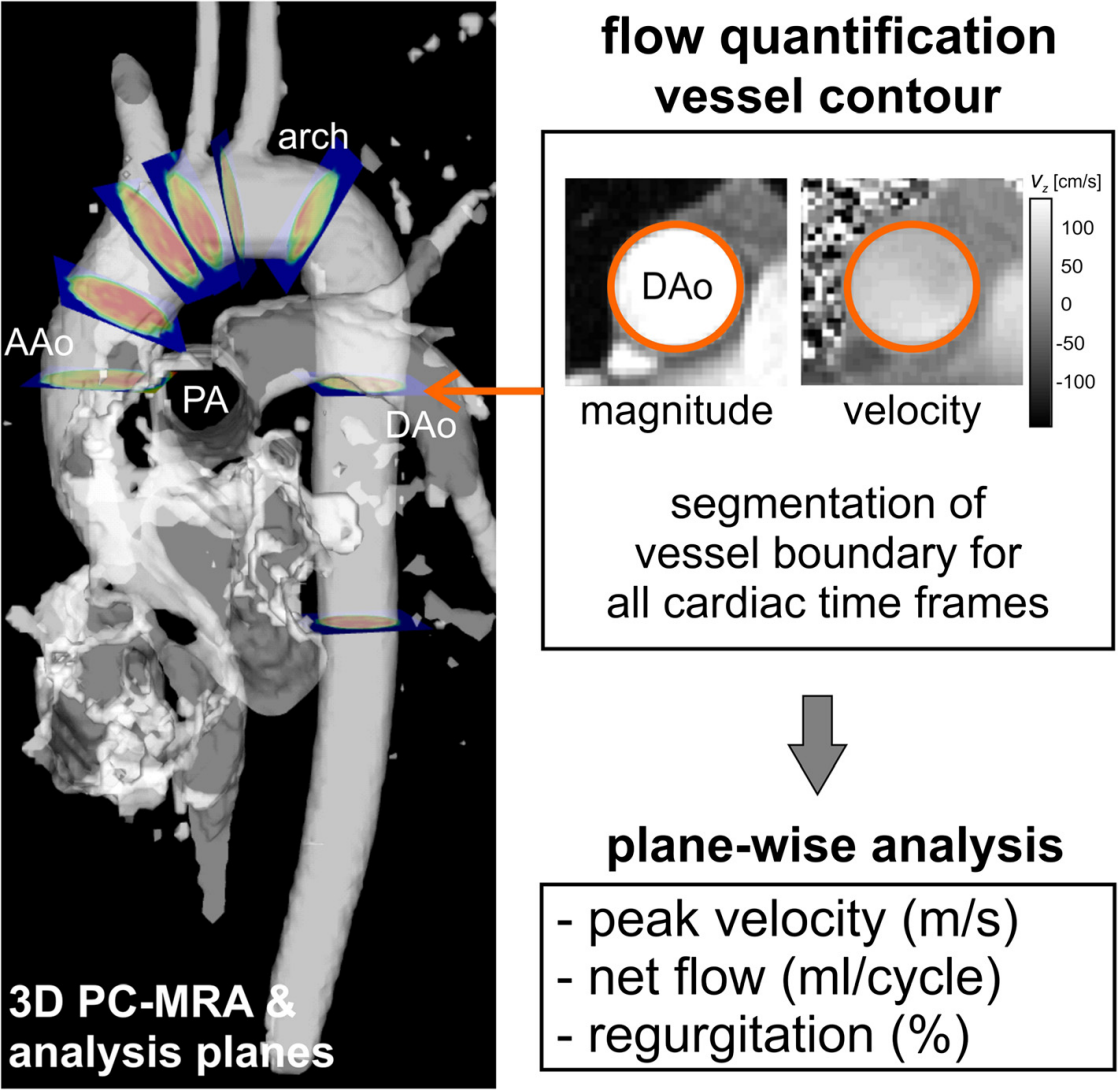
Illustration of retrospective flow quantification. For retrospective quantification of flow parameters based on 2D analysis, planes can be positioned at any anatomic location. In this example, an isosurface of 3D PC-MRA data derived from the 4D Flow CMR data (gray shaded) has been used to guide positioning analysis planes throughout the thoracic aorta. For each analysis plane, the vessel contours are segmented for all cardiac time frames to calculate flow volume, peak velocity and retrograde fraction

Flow volume and retrograde flow quantification in 4D Flow CMR is similar to that used in conventional 2D cine PC-CMR. However, a few important differences exist. As mentioned above and illustrated in Fig. 4, the volumetric coverage of 4D Flow CMR offers retrospective positioning of planes for flow volume measurements at any location within the acquired data volume [37, 47, 49, 162–164]. The use of a 3D or 4D PC-MR angiogram derived from the 4D Flow CMR data is recommended for anatomical orientation and identification of cross-sectional analysis planes for flow quantification. This may be combined with streamlines or pathlines visualizations for further guidance of plane positioning. Segmentation of the lumen can be done with similar approaches as in 2D cine PC-CMR, but due to smaller inflow effects, the contrast between blood and surrounding tissue is inferior in 4D Flow CMR. Another option is to perform 3D or 4D segmentation during data processing and use this segmentation as geometrical boundaries in the flow volume calculation. This approach can be advantageous for quantification of peak velocity in an entire vessel segment rather than relying on 2D analysis planes which do not coincide with the location of the maximum systolic velocity. Studies with 2D PC-CMR have shown that at least 5–6 voxels across the vessel lumen are needed for accurate flow volume quantification [165].

#### Quality control

Quality control is important for every clinical and research study. The versatility of 4D Flow CMR allows several approaches to be used, that can be included in imaging and post-processing without excessive additional effort.

Screening of 4D Flow CMR source images can reveal phase wraps, background phase offsets (by using narrow color-window), fold-over, and other image artifacts. Further, with its volumetric coverage, 4D Flow CMR offers several opportunities for control of the internal data consistency. For flow volume quantification, the conservation-of-mass principle can be employed to assess pulmonary vs. systemic flow volume ratios and flow volume in vs. out of the left ventricle [32, 38, 41–49]. The conservation-of-mass principle can also be used for quality control of pathlines analysis as the number of pathlines that enter and leave a specified region of interest should be the same (e.g. cardiac ventricles) [57]. Another complementary approach is to screen data for streamlines or pathlines that abruptly change direction or slowly drift out of the lumen, which can be indicative of phase wraps or uncompensated background phase offsets, respectively. Similarly, the presence of uncompensated background phase offsets can be suspected if pathlines emitted from the chest or back move in a non-random fashion.

The following approaches are recommended for general data quality control:

1. Visual inspection of source images
2. Quantitative quality control that targets the parameter of interest. For example, the conservation-of-mass principle is an excellent option when assessing the quality of flow volume quantification. We emphasize that requirements on the data depend on the analysis approach; sufficient data quality for accurate estimation of parameter A (e.g. peak velocity) does not necessarily imply accurate estimation of parameter B (e.g. flow volume). When the quantitative quality control method matches the analysis parameter of interest, this may be used as the first-in-line quality control step.
3. When the quantitative quality control signals poor data quality, we recommend performing additional visual inspection of the source images, as well as inspection of pathlines emitted from static tissue such as the chest or back.

### Controversies and recommendations for future work

The imaging sequences as well as data processing and analysis methods described in the recommendations section above constitute the current state of the technique as it is available at a large number of institutions. However, the field of 4D Flow CMR is rapidly evolving with improvements in imaging acquisition methods as well as data processing and analysis techniques. These development efforts increase the diversity of 4D Flow CMR. We encourage this trend, but we also see a need for improved conformity across sites and companies that develop and use 4D Flow CMR methods. There are also several limitations of current and emerging methods, some of which are not fully understood, and it is important to acknowledge the limitations and develop our understanding of them, so that improvements can be made where possible. This section outlines some of the more advanced techniques and areas for development in improving data quality and simplifying wide-scale clinical applicability.

### Advanced analysis parameters

Beyond the basic flow parameters discussed in the recommendation section above, a variety of more advanced analysis parameters are currently used in research settings, including wall shear stress, pressure difference mapping, kinetic energy, turbulent kinetic energy, energy dissipation, differential flow analysis, flow angles, flow displacement, and pulse wave velocity [40, 57, 59, 60, 103, 122, 123, 125–139, 166–168]. The quantitative information that these parameters provide can distinguish normal from abnormal blood flow, as well as differentiating types of abnormal hemodynamics. However, the application of these parameters can be complex. The effects of underlying assumptions, the impact of the quality of 4D Flow CMR data on the parameter, and the physiological meaning of the parameter as estimated with 4D Flow CMR should be taken into consideration. While a complete list of analysis parameters is beyond the scope of this consensus document, Table 3 contains some of the most common parameters and describes what they are, potential applications, the controversies and unmet needs.

**Table 3:** Commonly used advanced analysis parameters

Target parameter	Description	Potential applications	Requirements and uncertainties
Wall Shear Stress (WSS) [93, 125, 126, 193]	Viscous shear forces of flowing blood acting tangentially to the vessel wall	Indicator for impact of flow alterations on endothelial cell and extracellular matrix function and risk for vessel wall remodelling	Dependent on spatial resolution. Relationship to actual WSS values are unclear [127, 173]. Limited longitudinal data that demonstrates its predictive value for risk stratification.
Pulse Wave Velocity (PWV) [132, 133, 168]	Propagation speed of systolic pressure pulse in the arterial system	Marker of arterial stiffness and predictive of cardiovascular disease.	Requires high temporal resolution. Sensitive to artifacts.
Turbulent Kinetic Energy (TKE) [134, 136, 137]	Energy content of turbulent flow and direction-independent measure of intensity of turbulent velocity fluctuations	Estimate of turbulence-related loss of energy or pressure. Indicator of impact of turbulent flow on blood constituents or vessel wall.	The effect of intravoxel mean velocity variations affects the estimation of low TKE values. Is based on information from signal magnitude data from each individual flow-encoding segment, which are usually not obtained in standard reconstructions.
Relative Pressure Fields [128, 129, 131]	Relative blood pressure field	Noninvasive estimation of pressure differences	Pressure field calculations based on MR velocity data do not take turbulence effects into account and do therefore not reflect turbulence-related pressure losses that occur in stenotic flows. Computation of pressure fields is associated with several pitfalls and a best strategy has not been established.
Volume and Kinetic Energy of Ventricular Flow Components or Compartments [56, 57, 59]	Separation of blood that transits heart chambers according to compartmental origin and fate	Indicator of ventricular dysfunction. Risk stratification and optimization and individualization of treatment heart failure	Pathlines used to map the transit of blood through the chambers accumulate errors that are inversely related to the quality of velocity data. Mixing effects are unknown.

### Understanding the limits of the technique

Further work is needed to understand the accuracy and precision of existing and new 4D Flow CMR methods, including sequences, reconstruction methods and analysis parameters. The assessment of spatiotemporal fidelity and noise propagation of image acquisition, reconstruction and analysis methods is of key importance. Besides localization in space and time, any bias or noise-related uncertainty requires careful consideration, as it not only depends on the CMR experiment but also on MR system settings and tuning as shown in a recent 2D PC-CMR study [155].

### Spatial and temporal resolution

The acquisition of 4D Flow CMR data is, in a certain sense, complete. All dimensions and directions of the cyclically changing flow field are covered, albeit with spatial and temporal resolution that does not resolve all features of the flow. If partial k-space acquisitions are used, the method used for reconstruction, e.g. zero-filling or Margosian/homodyne reconstruction, should be reported [169]. The method needs to be chosen with respect to its impact on the phase of the MR signal. While spatial resolution is typically quoted as the ratio of field-of-view to acquisition matrix, it needs to be emphasized that the effective spatial resolution can be less. Likewise, the ability to resolve temporal features of flow may not be appropriately captured by quoting the number of acquired heart phases and any methods for temporal interpolation or view sharing should be reported. Utilizing the concept of spatiotemporal point-spread function (PSF_xt_) or transfer function is recommended for detailed investigations of a method’s ability to portray information [170–173]. Choices of spatial and temporal resolution need to be made according to the degree of spatial localization and temporal bandwidth required to sufficiently describe, depict, and measure the flow feature of interest. We emphasize that resolution is driven by application, and recommendations for measuring parameters such as flow volume may not be sufficient for quantities such as wall shear stress or pulse wave velocity. Careful choices and investigations are required if quantities are derived from the measured velocity vector fields including spatial and temporal velocity derivatives as required for assessing wall shear stresses, relative pressure fields or pulse wave velocities, for example. Many parameters are directly affected by the choice of temporal and spatial resolution and therefore the impact of spatial and temporal resolution on the accuracy and precision of a given parameter should be considered. It is recommended to assess if a different resolution would produce a different result. As a way of avoiding direction-dependent estimates, the acquisition of isotropic voxels is recommended. If this is not possible, the effect of voxel anisotropy should be investigated.

### Mean flow and small-scale variation in velocity

The time-resolved velocity fields measured with 4D Flow CMR are *mean* velocity fields and should be viewed as such. Spatial averaging occurs over the spatial extent of the voxel, and each measured cardiac phase (time frame) represents flow fields effectively averaged (phase-averaged) over multiple cardiac cycles extending over several minutes. The spatiotemporal resolution and effective averaging over multiple cardiac cycles limits the size of the flow features that can be characterized with velocity mapping techniques. However, the measured *mean* velocity field is accurate and corresponds very well to the actual mean velocity field [174–176]. In disturbed and turbulent flows, a *fluctuating* velocity field is superimposed on the *mean* velocity field. These small-scale velocity fluctuations are thus not resolved by 2D or 4D Flow CMR velocity mapping. In fact, resolving all scales of velocity is not a realistic goal for 4D Flow CMR velocity mapping, as this would require <0.1 mm spatial resolution and <1 ms real-time temporal resolution. However, this aspect of flow can be addressed by a complementary 4D Flow CMR technique referred to as intravoxel velocity standard deviation (IVSD) mapping, or turbulence mapping. This technique, which can be viewed as a flow-analogue to diffusion-weighted imaging, is based on an MR signal model that describes the relationship between the amplitude (not phase) of the PC-CMR signal and the range of velocities that are present in a voxel. The IVSD mapping technique permits the estimation of the intensity of turbulent velocity fluctuations and turbulent kinetic energy in stenotic flows [135, 174, 176, 177]. Its application in flows with only minor fluctuations may be hampered by the fact that laminar flow effects such as shear also give rise to intravoxel velocity variations that contribute to the measured IVSD. However, this effect appears to be small compared to intravoxel velocity variations caused by unstable fluctuations [60, 135].

### Noise propagation and confidence

Noise remains a limitation of the technique. An important parameter with respect to noise is the venc parameter that determines the velocity sensitivity of a 4D Flow CMR acquisition. The VNR is inversely proportional to the venc. Consequently, for a given venc, the estimation of low blood flow velocities < < venc is less reliable compared to flow velocities closer to venc. This can particularly be a limiting factor for multi-purpose flow analysis (e.g. quantification of both high flow velocities in a stenotic aorta and low flow velocities in a cardiac shunt in the same patient). New sequences are under development that permit the use of two or more venc’s [137, 147, 178]. In addition, other strategies can be employed to maximize SNR and VNR. This includes optimizing the experimental setup, including main magnetic field strength and receive-coil instrumentation, as well as protocol modifications.

Further work is required to understand the impact of noise, and we recommend the method of pseudo replicas [179, 180] to study and assess SNR and VNR dependencies and noise propagation. Accordingly, different noise realizations of same statistics are added to the original MR raw data and image reconstruction or parameter calculation is repeated to provide confidence intervals of velocity values. In a similar fashion, post-processing strategies including the impact of region-of-interest analysis can be tested and referenced.

### Systematic errors

Systematic errors causing unwanted bias of the measured velocity field are typically related to gradient induced eddy-currents, concomitant gradient fields and gradient non-linearity [148–151, 154]. While the latter two sources of error are corrected/calibrated with sufficient accuracy by clinical MR systems, eddy-currents depend on a range of parameters including the pre-emphasis settings of an individual MR system, gradient performance, orientation of the image volume and temperature of the gradient mount. Accordingly, prediction of the bias is often impossible and correction methods need to be applied retrospectively during image post-processing.

It is recommended to carefully study potential bias in a static gel phantom under identical experimental conditions, including navigators. This includes analysis of the spatial order of eddy-current induced background phase errors for each acquired heart phase [181]. If phantom calibration is applied routinely to subtract potential background phase offsets, fitted functions should be used to avoid compromising SNR/VNR of the original data upon subtraction. If background phase errors are corrected for by fitting polynomials through the phase of tissue known to be static, the fit error needs to be weighted against the degrees of freedom of the fit function to avoid over- or underfitting. Assessment of heart-phase dependent differences is recommended.

### Validation

In-vivo comparison against current gold-standard methods is lacking for many areas of 4D Flow CMR, often due to the lack of such a gold standard for in-vivo assessment. The entire chain of data acquisition, reconstruction and image processing should, if possible, be evaluated for accuracy and precision. It is helpful to compare with existing techniques, where they exist. However, 4D Flow CMR may provide more accurate quantification and so potentially become the new gold-standard, or it may be the only technique capable of assessing certain parameters. For areas where an in-vivo gold-standard is lacking, controlled steady and pulsatile flow phantom experiments with accurate reference quantification can be used to assess accuracy. In view of the range of commercial and custom-built phantoms available, it should be feasible to validate applications by simulating flow rates and pulsatility (e.g. Reynolds and Womersley number), cycle-to-cycle variation and presence of sufficient static tissue for background correction. Reference methods (Particle Tracking or Image Velocimetry, Laser Doppler Anemometry) can also be used to establish baseline data in-vitro. Numerical phantoms providing idealized model data are important for the study of certain aspects of data reconstruction and processing in flow fields that are fully known. Evaluation of precision should include testing sensitivity especially to spatiotemporal resolution and SNR, which can be done in-vivo, in-vitro, or using simulations.

### Status of implementation and standardization

#### Sequence

In addition to the sequence settings described in Table 2, several options exist for non-Cartesian 4D Flow CMR [182–186]. Different acquisition strategies (Cartesian, spiral, radial, EPI, bSSFP, etc.) have different strengths and weaknesses and thus the optimal acquisition strategy depends on the targeted application and analysis parameter. Moreover, in addition to standard parallel imaging, more advanced acceleration techniques have shown promising results and there exists many options for reduction of 4D Flow CMR scan times [39, 49, 187–192]. Reduced scan times are particularly relevant to applications in smaller vessels where higher resolution is needed.

At the time of writing, none of the major MR systems manufacturers (GE, Philips, Siemens) routinely provide 4D Flow CMR sequences or packages to researchers or clinical users. On Philips scanners, however, the necessary sequence exists and users can set up a 4D Flow CMR protocol (‘exam card’), similar to the consensus protocol, on a standard commercial system without any software modifications. Siemens offer a ‘work-in-progress’ package to selected users. Due to the lack of widely available commercial 4D Flow CMR sequences, a large number of studies and applications are still based on 4D Flow pulse sequences that individual research groups have developed in-house and shared with collaborators worldwide. 4D Flow CMR pulse sequences implemented by research groups exist in a variety of flavors (different k-space trajectories, acceleration methods, etc.) and for all major MR platforms (GE, Philips, Siemens). The lack of standardization across MR platforms (even for the same vendor) and data output formats, as well as the absence of commercial 4D Flow CMR sequences and protocols are limiting factors that hinder introduction of the technique to the clinical environment.

#### Software for pre-processing, visualization and flow quantification

Pre-processing, visualization and flow quantification is being performed using in-house developed tools, early-stage commercial packages, or manufacturer prototypes. The field would benefit from greater standardization in data analysis methods, workflows, and data output formats, which in turn affect the use of analysis tools. Wide clinical utility would benefit from the availability of user-friendly tools that are integrated in MR scanner consoles and workstations, as well as PACS systems. This would ideally include the following capabilities: 1) retrospective flow quantification on the scanner console and/or workstations and/or PACS system, 2) analysis and representation of clinically relevant parameters such as flow waveforms and cardiac output in DICOM format 3) 4D Flow visualizations on MR scanner console and/or workstations and/or PACS systems, and 4) animations in DICOM format to store and display in PACS. We encourage vendors and third-party developers to consider implementing these key features as a basis for more routine clinical use of 4D Flow CMR.

## Conclusion

Relatively easy scan prescription and retrospective placement of analysis planes makes 4D Flow CMR a potentially advantageous tool in the clinical setting, particularly if several regions and directions of flow merit investigation. Conventional flow parameters can be obtained at any location in the data volume where the employed parameter settings provide sufficient accuracy. At the same time, 4D Flow CMR visualizations offer more versatile and comprehensive depictions of flow fields than any other in-vivo imaging technique. Further, advanced 4D Flow CMR analysis parameters are currently used in the research setting but require testing for clinical utility. Widespread clinical usage would be facilitated by further integration into the standard MR environment. Multicenter studies are necessary to establish the repeatability of various aspects of the technique across centers.
